# Hierarchical integration of DNA nanostructures and NanoGold onto a microchip facilitates covalent chemistry-mediated purification of circulating tumor cells in head and neck squamous cell carcinoma

**DOI:** 10.1016/j.nantod.2023.101786

**Published:** 2023-02-20

**Authors:** Na Sun, Ceng Zhang, Jing Wang, Xinmin Yue, Hyo Yong Kim, Ryan Y. Zhang, Hongtao Liu, Josephine Widjaja, Hubert Tang, Tiffany X. Zhang, Jinglei Ye, Audrey Qian, Chensong Liu, Alex Wu, Katharina Wang, Michael Johanis, Peng Yang, Honggang Liu, Meng Meng, Li Liang, Renjun Pei, Wanxing Chai-Ho, Yazhen Zhu, Hsian-Rong Tseng

**Affiliations:** a California NanoSystems Institute, Crump Institute for Molecular Imaging, Department of Molecular and Medical Pharmacology, University of California, Los Angeles, Los Angeles, CA 90095, USA; b Key Laboratory for Nano-Bio Interface, Suzhou Institute of Nano-Tech and Nano-Bionics, University of Chinese Academy of Sciences, Chinese Academy of Sciences, Suzhou 215123, China; c Department of Pathology, School of Basic Medical Sciences, Southern Medical University, Guangzhou 510515, China; d Department of Pathology, Beijing Tongren Hospital, Capital Medical University, Beijing 100730, China; e College of Pharmacy, State Key Laboratory of Medicinal Chemical Biology and Tianjin Key Laboratory of Molecular Drug Research, Nankai University, Tianjin 300353, China; f Department of Pathology, The First Affiliated Hospital of Shandong First Medical University & Shandong Provincial Qianfoshan Hospital, Shandong 250014, China; g Department of Pathology, Nanfang Hospital and School of Basic Medical Sciences, Southern Medical University, Guangzhou 510515, China; h Guangdong Province Key Laboratory of Molecular Tumor Pathology, Guangzhou 510515, Guangdong Province, China; i Department of Medicine, Division of Hematology/Oncology, David Geffen School of Medicine, University of California, Los Angeles, Los Angeles, CA 90095, USA; j Jonsson Comprehensive Cancer Center, University of California, Los Angeles, Los Angeles, CA 90095, USA; k Department of Pathology and Laboratory Medicine, David Geffen School of Medicine, University of California, Los Angeles, Los Angeles, CA 90095, USA

**Keywords:** NanoGold, Tetrahedral DNA nanostructure, Covalent chemistry, Circulating tumor cell, Head and neck squamous cell carcinoma

## Abstract

It is well-established that the combined use of nanostructured substrates and immunoaffinity agents can enhance the cell-capture performance of the substrates, thus offering a practical solution to effectively capture circulating tumor cells (CTCs) in peripheral blood. Developing along this strategy, this study first demonstrated a top-down approach for the fabrication of tetrahedral DNA nanostructure (TDN)-NanoGold substrates through the hierarchical integration of three functional constituents at various length-scales: a macroscale glass slide, sub-microscale self-organized NanoGold, and nanoscale self-assembled TDN. The TDN-NanoGold substrates were then assembled with microfluidic chaotic mixers to give TDN-NanoGold Click Chips. In conjunction with the use of copper (Cu)-catalyzed azide-alkyne cycloaddition (CuAAC)-mediated CTC capture and restriction enzyme-triggered CTC release, TDN-NanoGold Click Chips allow for effective enumeration and purification of CTCs with intact cell morphologies and preserved molecular integrity. To evaluate the clinical utility of TDN-NanoGold Click Chips, we used these devices to isolate and purify CTCs from patients with human papillomavirus (HPV)-positive (+) head and neck squamous cell carcinoma (HNSCC). The purified HPV(+) HNSCC CTCs were then subjected to RT-ddPCR testing, allowing for detection of E6/E7 oncogenes, the characteristic molecular signatures of HPV(+) HNSCC. We found that the resulting HPV(+) HNSCC CTC counts and E6/E7 transcript copy numbers are correlated with the treatment responses in the patients, suggesting the potential clinical utility of TDN-NanoGold Click Chips for non-invasive diagnostic applications of HPV(+) HNSCC.

## Introduction

Studies in the past decades have documented that nanoscale structures located in the tissue microenvironment, such as the cell surface (e.g., microvilli and filopodia) [1] and the extracellular matrix (ECM) [2], serve as structural and biochemical supports that play pivotal roles in regulating cellular behaviors, fates, and functions (e.g., morphology, adhesion, viability, migration, and differentiation). [3–5] Inspired by the crucial role of nanoscale interactions in the tissue microenvironment, the development of nanostructure-embedded substrates [6], which mimic nanoscale constituents, has attracted significant attention in the interface [7,8] of nanotechnology and cancer diagnostics. Given that their nanostructured surface can enhance immuno-affinity to the cell surface, these substrates offer a simple solution to efficiently enrich the rare cells [9] in biological samples, such as blood. By taking advantage of this unique feature, we and others have pioneered the use of nanostructured substrates for enrichment, isolation, and characterization of rare cells, including, but not limited to, circulating tumor cells (CTCs) [6] and circulating fetal nucleated cells (CFNCs) [10–12]. Particularly, NanoVelcro Chips [13–15] developed by our research team have been applied to various clinical needs such as CTC enumeration [16], single-cell analysis [17], and CTC purification [18,19] with well-preserved RNA and cell viability. Though it has been successfully demonstrated that the previously developed nanostructured substrates enrich rare cells by relying on the nanostructure-mediated cell-affinity capture, all of these methods are based on the integration of a single layer of nanostructure onto a supporting substrate.

**Fig. 1.**
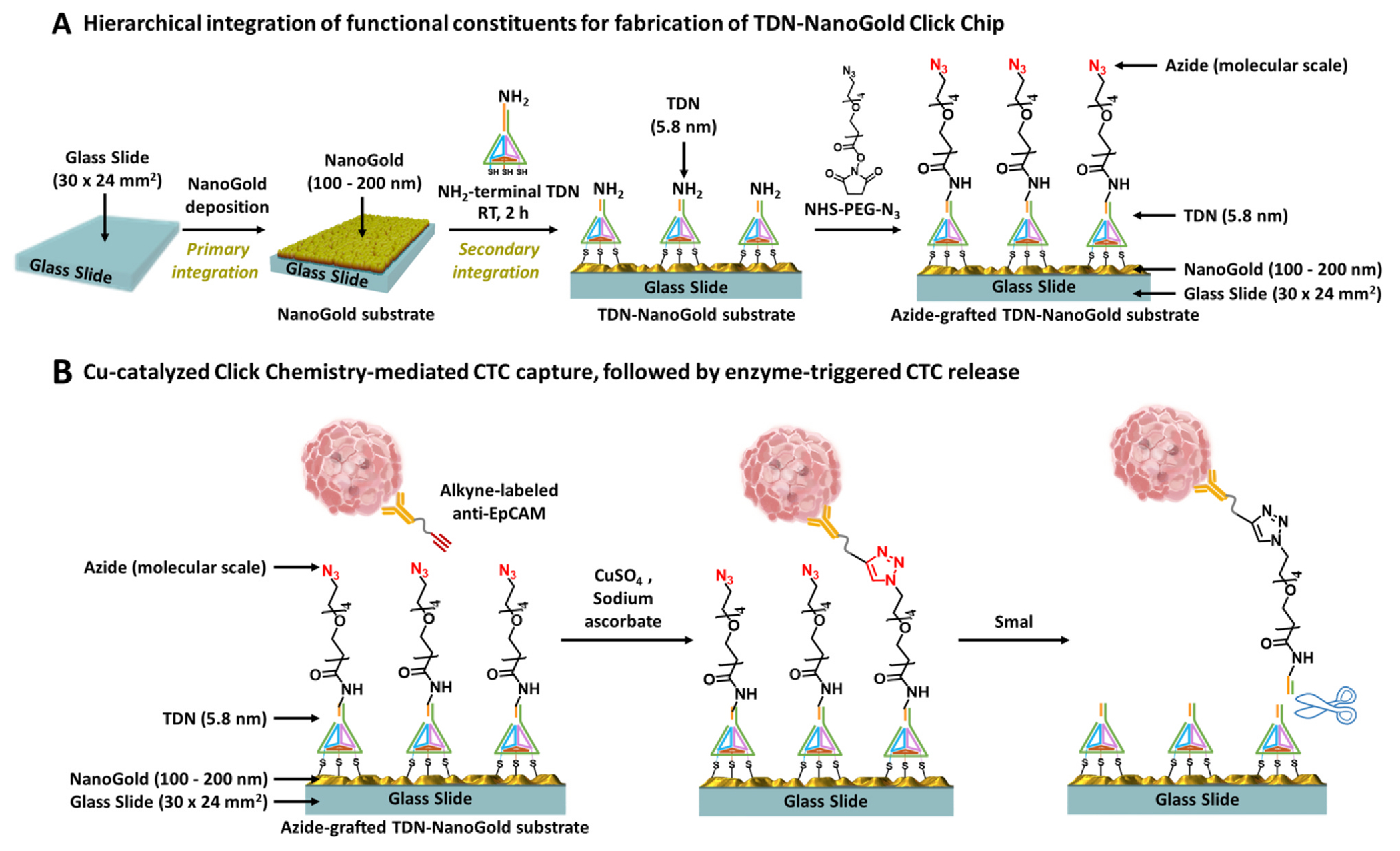
A) Fabrication of tetrahedral DNA nanostructures (TDN)-NanoGold substrate via hierarchical integration of functional constituents. A glass slide (macroscale; 30 × 24 mm^2^) was used as the supporting substrate, onto which self-organized gold substrates (sub-micro; 100–200 nm) were deposited. Self-assembled TDN were then layered on gold substrates, generating a TDN-NanoGold substrate. Finally, azide motifs were covalently grafted onto the TDN-NanoGold substrate to give an azide-grafted TDN-NanoGold substrate. B) Copper (Cu)-catalyzed azide-alkyne cycloaddition (CuAAC)-mediated capture of circulating tumor cells (CTCs), followed by enzyme-triggered release to purify CTCs. Alkyne-grafted anti-EpCAM was first conjugated onto CTCs. In the presence of Cu(I), CuAAC resulted in the immobilization of CTCs onto TDN-NanoGold substrate. After CuAAC-mediated CTC capture, a restriction enzyme, *Sma*I, was employed to specifically cleave the targeted sequence in TDN to release CTCs.

Herein, we introduce a novel fabrication approach for a nanostructure-embedded rare-cell assay, i.e., tetrahedral DNA nanostructures (TDN)-NanoGold Click Chips, through the hierarchical integration of three functional constituents with multiple length-scales. As depicted in Fig. 1 A, TDN-NanoGold Click Chips were constructed by sequentially layering constituents through a top-down approach, from macro to sub-micro to nano to molecular scales. First, a glass slide (macroscale; 30 × 24 mm^2^) served as the supporting substrate, on which the primary integration of self-organized gold structures (sub-microscale; 100–200 nm) was performed by a gold seeding approach [20], resulting in distinct surface morphology of gold nanoislands, a.k.a., NanoGold substrate. Subsequently, the secondary integration of self-assembled TDN (nanoscale; 5.8 nm) onto NanoGold structures was conducted via a thiol-gold redox reaction [21], resulting in TDN-NanoGold substrates. Finally, a Click Chemistry motif, azide (N_3_), was covalently grafted onto the substrates to produce an azide-grafted TDN-Nano-Gold substrate. After incorporating an overlaid polydimethylsiloxane (PDMS) chaotic mixer [13], the resultant TDN-NanoGold Click Chip can be used for the efficient capture of CTCs. Our proof-of-concept study demonstrated that all three functional constituents (i.e., macroscale glass slide, sub-microscale NanoGold, and nanoscale TDN) integrated in the TDN-NanoGold substrates (Fig. 1A) are essential to achieving high-affinity cell capture.

Click chemistry [22] is a class of rapid bio-orthogonal organic reactions frequently used for bioconjugation. Given the fact that there may be a low number of antigens present on the surface of individual CTCs (due to epithelial-mesenchymal transition), conventional immunoaffinity-based CTC capture approaches could suffer from poor capture performance. Our previous research demonstrated [18] that this issue can be overcome by replacing the immunoaffinity-mediated CTC capture with click chemistry-mediated CTC capture, where the inverse-electron-demand Diels-Alder cycloaddition [23] between tetrazine (Tz) and TCO motifs was used. The click chemistry reaction between Tz-grafted CTC Click Chips and TCO-grafted CTCs is rapid, specific, and irreversible, leading to the immobilization of CTCs onto Click Chip [18] with improved capture efficiency and reduced background signals. However, the tradeoff of incorporating highly reactive Tz and TCO motifs for enhanced CTC capture is the short shelf life of the Tz-modified Click Chips and TCO-modified antibody, which limits their long-term storage and utility. To address this technical challenge, we explored the use of copper (Cu)-catalyzed azide-alkyne cycloaddition (CuAAC) [24–26] (Fig. 1B). In contrast to previous Tz-TCO Click Chemistry-mediated cell capture, the CuAAC motifs are relatively stable yet sufficiently reactive in the presence of Cu(I), generated in situ by mixing CuSO_4_ and sodium ascorbate. Here, we introduced an azide motif onto the TDN-NanoGold substrate and modified an anti-EpCAM capture agent with an alkyne motif. After CuAAC-mediated cell capture, a restriction enzyme, *Sma*I, [27,28] was employed to specifically cleave the targeted sequence in TDN, leading to higher CTC recovery with intact cell morphologies and preserved molecular integrity. This innovation allowed us to improve CTC capture performance while lengthening the lifetime of Click Chips [18].

**Fig. 2.**
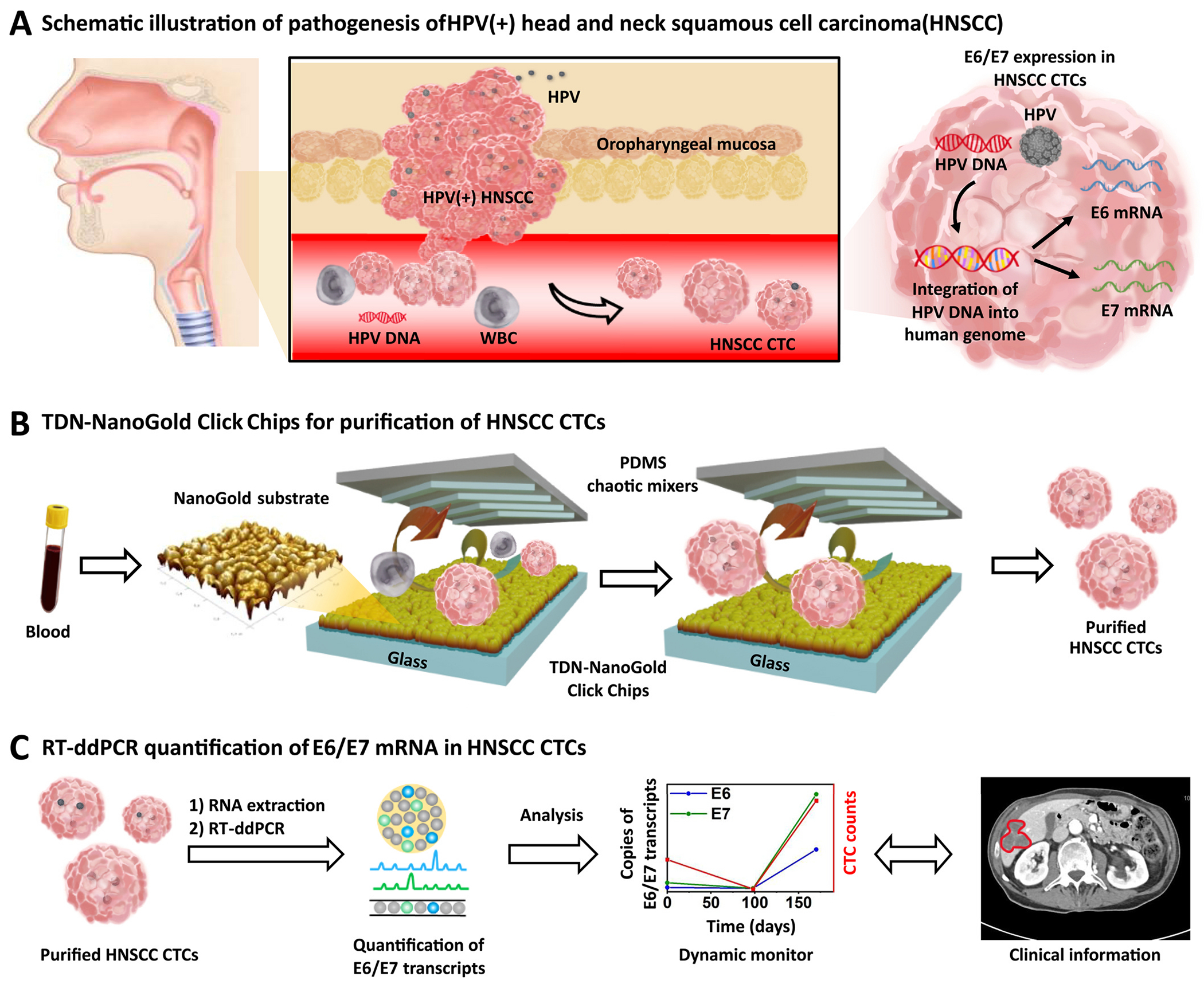
TDN-NanoGold Click Chips for purification of HNSCC CTCs, followed by quantification of E6/E7 transcripts in the purified HNSCC CTCs. A) HPV infection of oropharyngeal mucosa in the upper respiratory tract may lead to the development of HNSCC due to the integration of HPV DNA into the host genome. The expression of E6/E7 transcripts is a characteristic molecular signature of HPV DNA integration and can be found in HNSCC CTCs when they are shed into blood circulation. B) Schematic illustration of TDN-NanoGold Click Chips featuring two functional components: an azide-grafted TDN-NanoGold substrate and an overlaid PDMS chaotic mixer for purification of HNSCC CTCs. C) The purified HNSCC CTCs are subjected to RT-ddPCR to quantify the copy numbers of E6/E7 transcripts.

Head and neck squamous cell carcinoma (HNSCC) [29,30] is the sixth most common malignancy worldwide. Human papillomavirus (HPV) has emerged as a novel risk factor (Fig. 2A) for HNSCC, defining a new subtype of tumor that is distinct from HPV(−) ones. [31–33] The most prominent type of HPV, HPV16, accounts for approximately 90% of HPV(+) HNSCC cases. Its genome contains the E6 and E7 oncogenes, whose transcription levels are characteristic molecular signatures of HPV(+) HNSCC. Since HPV infects and replicates in a fully differentiating squamous epithelium only, [34] the E6 and E7 oncogenes can only be derived from epithelial cells rather than from leukocytes in circulation. This unique characteristic makes CTC-derived E6/E7 oncogenes a promising specific liquid biopsy marker for quantification of the systemic tumor burden. It is widely accepted that the P16 protein detected by immunohistochemistry (IHC) is a surrogate marker for HPV infection. [35] Therefore, we evaluated the feasibility of using TDN-NanoGold Click Chips to isolate and purify HNSCC CTCs and their P16(+) sub-populations that have been shed into circulation, followed by quantification of CTC-derived E6/E7 oncogenes. Fourteen blood samples were collected from 11 HPV(+) HNSCC patients for CTC enumeration studies using TDN-NanoGold Click Chips, which consists of an azide-grafted TDN-NanoGold substrate and an overlaid PDMS chaotic mixer (Fig. 2B), enabling highly effective CuAAC-mediated capture of HNSCC CTCs. After this Click Chemistry-mediated capture and the enzyme-triggered release of the CTCs, the purified HNSCC CTCs were subjected to RNA extraction, followed by quantification of E6/E7 transcripts by reverse transcription-droplet digital PCR (RT-ddPCR). By analyzing the dynamic changes in CTC counts and E6/E7 transcript quantification in conjunction with computed tomography (CT) images, we found that the resulting CTC counts and E6/E7 transcript copy numbers are strongly correlated with the treatment responses in the patients, which demonstrates the clinical utility of TDN-NanoGold Click Chips in the noninvasive diagnosis of HPV(+) HNSCC (Fig. 2C).

## Results and discussion

### The preparation and characterization of a TDN-NanoGold Click Chip

A TDN-NanoGold Click Chip is composed of two functional components: (i) an azide-grafted TDN-NanoGold substrate and (ii) an overlaid PDMS chaotic mixer housed in a custom-designed microfluidic chip holder. The NanoGold substrate was formed by introducing self-organized NanoGold structures onto a glass substrate via a gold seeding approach and then characterized using a scanning electron microscope (SEM) and a Bruker atomic force microscope (AFM) (Fig. S1). The sizes of these self-organized gold islands range between 100 and 200 nm, which provides increased substrate surface contact area to improve capture and recovery yield. [36,37] To form the TDN-NanoGold substrate, self-assembled TDN were grafted onto the NanoGold substrate via a thiol-gold redox reaction. Based on the concerns about assembly efficiency, stability and effective conjugating surface, the TDN with the size of 5.8 nm were prepared onto the nano-gold in this study. [38–40] The design of DNA sequences used in TDN is provided in Table S1. In addition, the preparation and characterization of TDN and its *Sma*I-triggered cleavage are investigated in Fig. S2 and S3, respectively. The conjugation of TDN was conducted by mixing all DNA strands, followed by denaturation at 95 °C for 10 min and annealing at 4 °C (Fig. S2A). This successful step-by-step TDN self-assembly was monitored and characterized by polyacrylamide gel electrophoresis (PAGE) (Fig. S2B) and confirmed via the dynamic light scattering (DLS) and AFM analyses (Fig. S2C and S2D). Furthermore, the specific cleavage of the target TDN sequence by *Sma*I was confirmed by PAGE (Fig. S3).

**Fig. 3.**
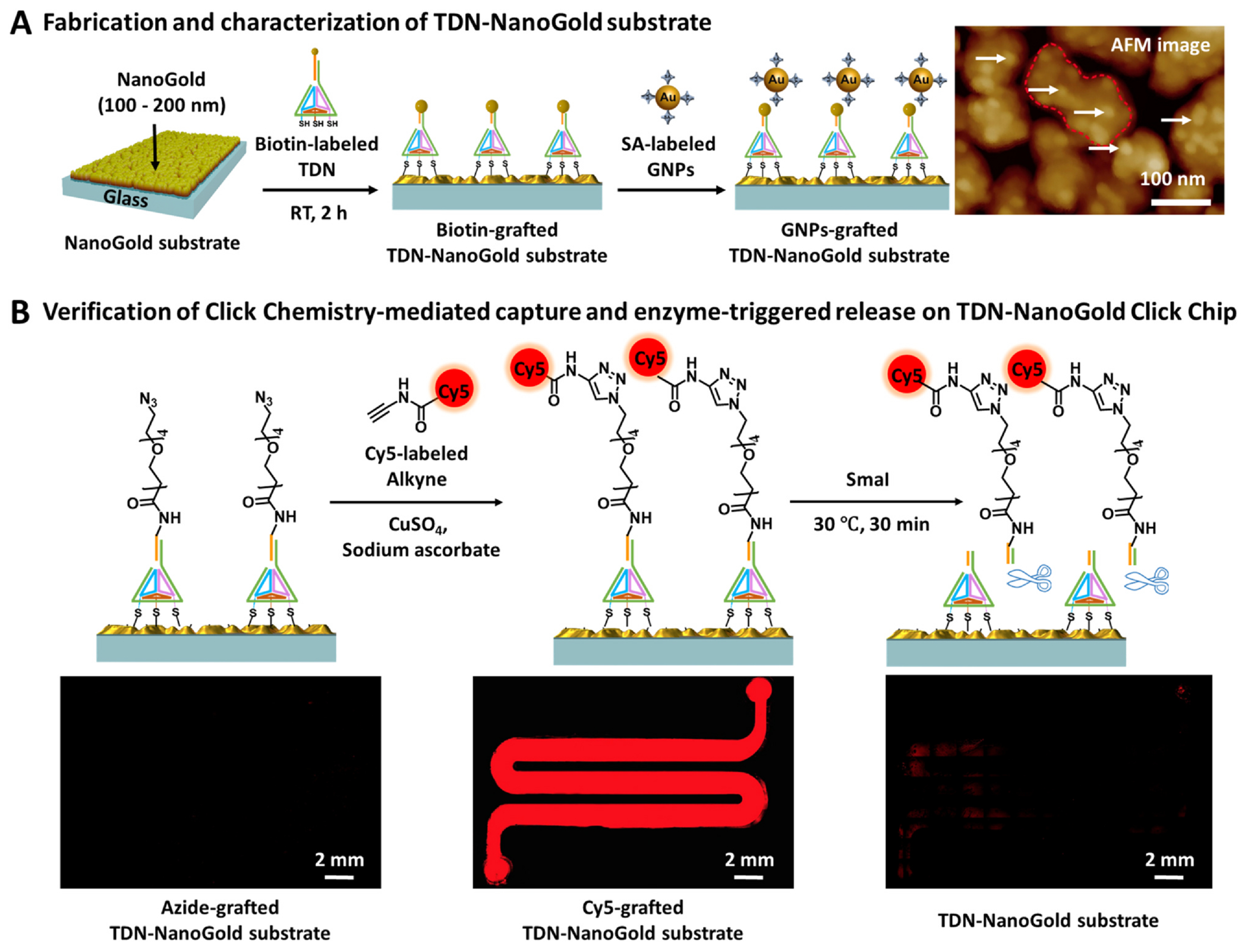
Characterization of TDN-NanoGold Click Chips. A) Fabrication and characterization of TDN-NanoGold substrate. A biotin-grafted TDN-NanoGold substrate was prepared via self-assembly of biotin-grafted TDN onto NanoGold substrate. After incubation with SA-labeled GNPs (10 nm), the resultant GNPs-grafted TDN-NanoGold substrate was characterized by AFM. Densely packed GNPs were observed on the gold nanoislands of the NanoGold substrate. B) Verification of CuAAC-mediated capture and enzyme-triggered release on TDN-NanoGold Click Chip. The CuAAC reaction between Cy5-labeled alkyne and azide-grafted TDN-NanoGold substrate led to immobilization of Cy5 as shown in the fluorescence micrograph (Nikon 90i; λ_ex_ = 620 nm and λ_em_ = 670 nm). Cy5 was released after enzyme-triggered cleavage by *Sma*I, resulting in dramatically reduced fluorescence signals.

In the top-down fabrication of the TDN-NanoGold substrate, biotin-labeled TDN was designed and grafted onto the NanoGold substrate, followed by incubation with streptavidin (SA)-labeled gold nanoparticles (GNPs; 10 nm). The AFM image of the resultant GNPs-grafted TDN-NanoGold substrate (Fig. 3A) showed that GNPs were evenly distributed on the surface of the substrate, implying the successful fabrication of the TDN-NanoGold substrate. We also verified the successful preparation of azide-grafted TDN-NanoGold substrates via the CuAAC reaction between Cy5-labeled alkyne and azide-grafted TDN-NanoGold substrate, followed by an enzyme-triggered cleavage of TDN by *Sma*I (Fig. 3B). First, the solution of Cy5-labeled alkyne was run through the TDN-NanoGold Click Chip, which allowed Cy5-labeled alkyne to be grafted onto azide motifs on the substrate, generating a strong red fluorescent signal along the serpentine microfluidic channel. After exposure to *Sma*I, the red fluorescent signal was significantly reduced, verifying the enzyme-triggered release of the grafted Cy5 fluorescent dye. These experiments demonstrated the synergy of the CuAAC-mediated capture and enzyme-triggered release in TDN-NanoGold Click Chips.

### Optimization of TDN-NanoGold Click Chips for CuAAC-mediated capture and enzyme-triggered release of HNSCC CTCs

To accurately evaluate the performance of TDN-NanoGold Click Chips throughout the optimization process, artificial plasma samples were prepared by spiking fifty SCC090 cells, an HPV16(+) HNSCC cell line, into peripheral blood mononuclear cells (PBMCs) isolated from whole blood of a healthy donor (2 mL). As SCC090 cells harbor HPV genes (i.e., E6), which are absent in healthy donor’s blood, SCC090 cells can be specifically quantified by detecting E6 transcript.

**Fig. 4.**
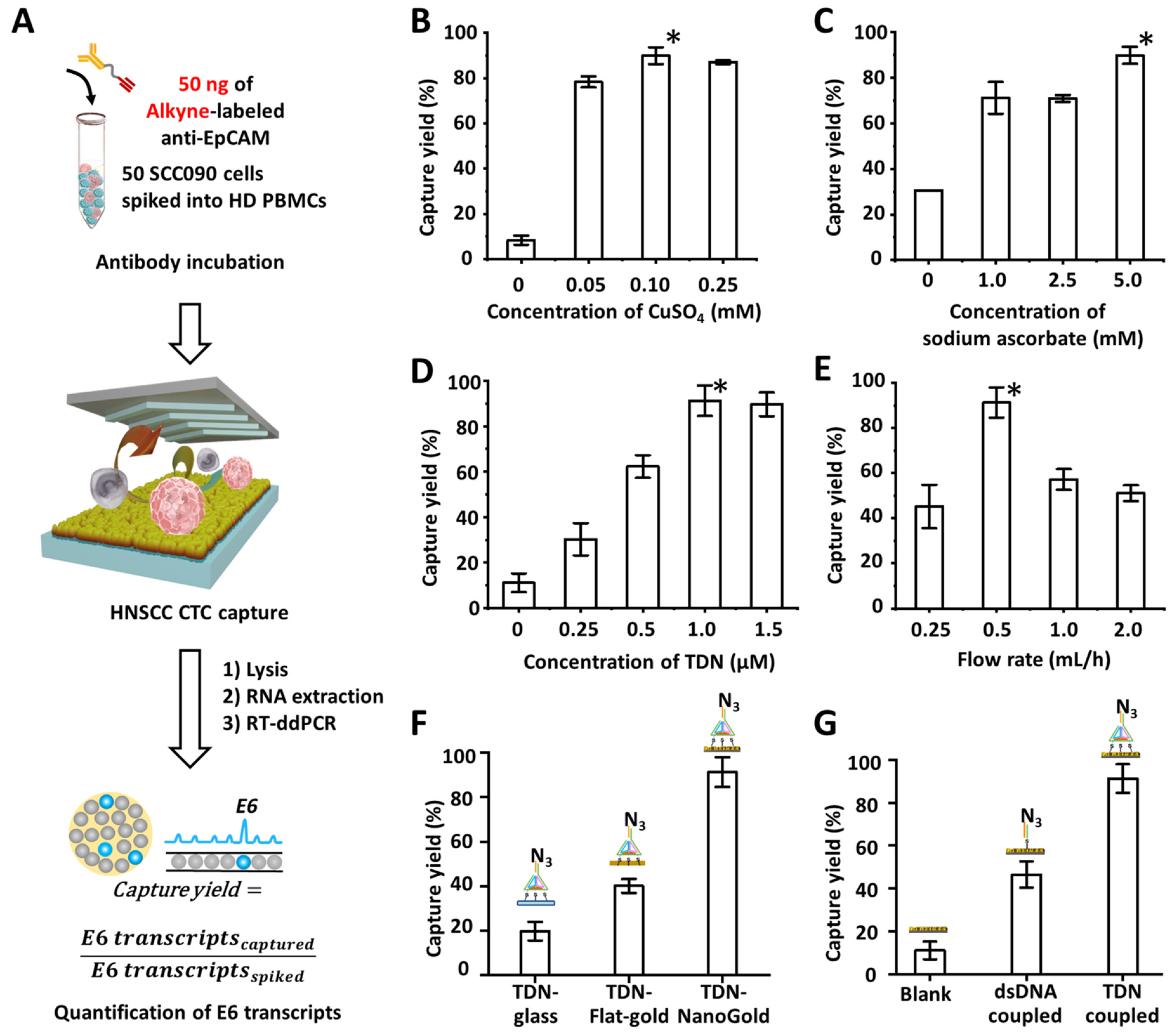
Optimization of TDN-NanoGold Click Chips for CuAAC-mediated capture of HNSCC CTCs. A) A general workflow for optimization of TDN-NanoGold Click Chips for CuAAC-mediated capture of HNSCC CTCs using artificial samples. After CuAAC-mediated capture of HNSCC CTCs and subsequent lysis, E6 transcripts were quantified by RT-ddPCR, allowing the calculation of capture yields. We investigated the HNSCC CTC capture yields as a function of B) concentration of CuSO_4_ (mM), C) concentration of sodium ascorbate (mM), D) concentration of TDN (μM), and E) flow rate (mL/h). We also demonstrated the necessity of hierarchical integration of both NanoGold and TDN by comparing CTC capture yields among F) azide-grafted TDN-glass substrate, azide-grafted TDN-flat-gold substrate, and TDN-NanoGold Click Chips, and G) blank NanoGold substrate, azide-grafted dsDNA-NanoGold substrate, and TDN-NanoGold Click Chips.

We first optimized the HNSCC CTC capture yield. As shown in the workflow depicted in Fig. 4A, a TDN-NanoGold Click Chip was used to capture CTCs. The individual Alkyne-conjugated antibody was prepared just before use (Fig. S4). Then, the immobilized CTCs were lysed in the devices. After extracting mRNA from the lysed mixtures, the E6 transcript was quantified by RT-ddPCR. The capture yields for SCC090 cells were calculated by Eq. (1) throughout the optimization process:

(1) $$\mathrm{Capture yield}=E6\;{\mathrm{transcripts}}_{\mathrm{captured}}/E6\;{\mathrm{transcripts}}_{\mathrm{spiked}}$$

We examined the experimental parameters for CuAAC-mediated CTC capture, including the concentrations of CuSO_4_ and sodium ascorbate. Various concentrations of CuSO_4_ (0, 0.05, 0.10, and 0.25 mM) and sodium ascorbate (0, 1.0, 2.5, and 5.0 mM) were tested, and the optimal concentrations were determined as 0.1 mM of CuSO_4_ and 5.0 mM of sodium ascorbate (Fig. 4B and C). With these conditions for the CuAAC reaction, the concentrations of TDN (0, 0.25, 0.5, 1.0, and 1.5 μM) for TDN-NanoGold substrate fabrication were also optimized, and a TDN concentration of 1.0 μM resulted in the highest capture yield of 91.3 ± 6.7% (Fig. 4D). The flow rates of the samples (0, 0.5, 1.0, and 2.0 mL/h) were examined, and the highest capture yield was observed at the flow rate of 0.5 mL/h (Fig. 4E).

To demonstrate the necessity of the hierarchical integration of both the NanoGold and TDN for the fabrication of TDN-NanoGold Click Chip, we conducted control experiments using devices that replaced the self-assembled TDN or self-organized NanoGold structures. First, the capture yields of the TDN-NanoGold Click Chip were compared to those of devices without the NanoGold constituent (TDN-glass) and with a flat-gold constituent (TDN-flat-gold). The TDN-NanoGold Click Chip exhibited a higher capture yield of 91.3 ± 6.7% (Fig. 4F), compared to the TDN-glass capture yield (19.7 ± 4.2%) and the TDN-flat-gold capture yield (40.1 ± 3.1%). To better understand how NanoGold substrate facilitates CTC capture, scanning electron microscopy (SEM) was employed to characterize the interfaces between captured cells and the TDN-NanoGold Click Chip. As a control, SEM was also performed in parallel using flat substrates without NanoGold structures. As shown in Fig. S5, many cellular protrusions (Fig. S5A, B) were observed between a captured cancer cell and the NanoGold substrate, while the cells on the flat substrate appear more rounded, and relatively fewer cellular protrusions (Fig. S5C) were observed. These results indicate that NanoGold structures improve capture efficiency through enhancing the interactions between cancer cells and substrates similar to the mechanisms described in earlier publications [9]. Then, the capture yields of the TDN-NanoGold Click Chip were compared to those of a NanoGold substrate without TDN (blank) and a NanoGold substrate with double-stranded DNA (dsDNA coupled). The TDN-NanoGold Click Chip generated the highest capture yield (91.3 ± 6.7%) compared to those observed for the controls: 11.1 ± 4.1% for blank and 46.5 ± 6.1% for dsDNA coupled (Fig. 4G). This improvement may result from the 3D nanostructure of TDN, which provides a more accessible space for alkyne motifs labeled on CTCs to come in contact with azide motifs grafted onto the substrate. Therefore, both primary and secondary integrations of the self-organized NanoGold structures and the self-assembled TDN, respectively, are crucial in achieving the enhanced performance of the TDN-NanoGold Click Chip.

We and others have demonstrated that incorporating nanostructures onto capture agent-coated substrates facilitates cell affinity [9]. The enhanced recovery yield can be attributed to the increased surface area available for contact between nanofeatures on the substrates and nanoscale cell-surface components, allowing for more binding sites to achieve higher capture efficiency. The hierarchical integration of the three functional constituents to fabricate TDN-NanoGold Click Chip offers a top-down fabrication approach for preparing nanostructured substrates. All three of the functional constituents enhance cell-substrate interactions, including 1) substrate surface contact area (sub-microscale NanoGold), 2) capture agent loading capacity (nanoscale TDN), and 3) other physical interactions of cellular surface components (i.e., filopodia or lamellipodia, as shown in Fig. S5), which significantly contribute to higher cell capture performances.

**Fig. 5.**
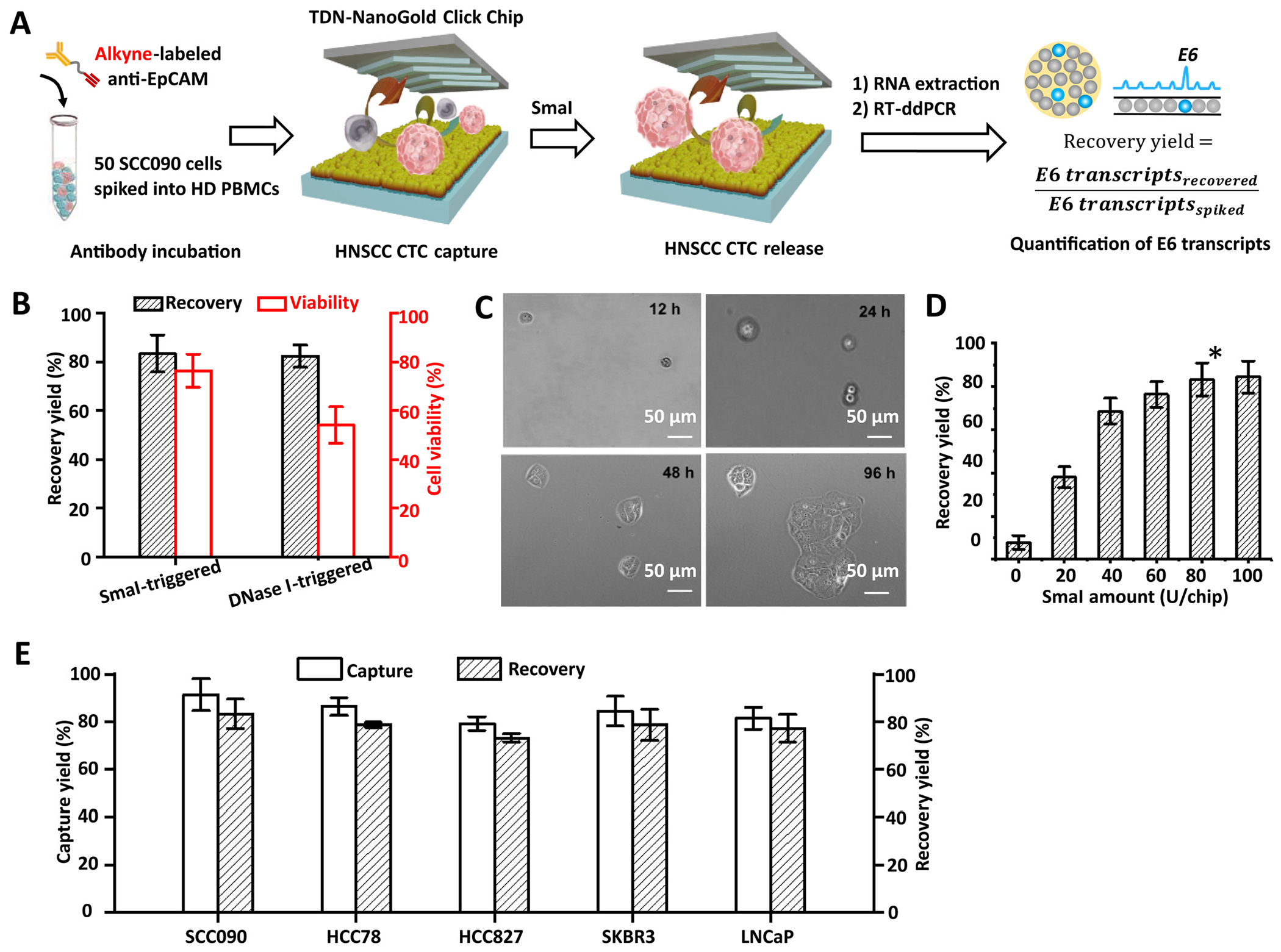
Optimization of TDN-NanoGold Click Chips for enzyme-triggered release of HNSCC CTCs. A) A general workflow for optimization of TDN-NanoGold Click Chips for enzyme-triggered release of HNSCC CTCs using artificial samples by RNA quantification. After CuAAC-mediated capture of HNSCC CTCs followed by their enzyme-triggered releases, E6 transcripts were quantified by RT-ddPCR, allowing calculation of recovery yields. B) Comparison of recovery yields and viabilities of CTCs recovered from the TDN-NanoGold Click Chips by employing specific restriction enzyme cleavage (*Sma*I; 80 U/chip) and non-specific endonuclease cleavage (DNase I; 60 U/chip). C) Micrographic images of the SCC090 cells for 12–96 h after their capture and release from TDN-NanoGold Click Chips. D) The recovery yields of TDN-NanoGold Click Chips according to the varying amounts of *Sma*I (0–100 U/chip) at a flow rate of 0.5 mL/h. * Indicates the optimal condition identified in this study. E) General applicability of the TDN-NanoGold Click Chips for CTC purification was validated by using artificial samples spiked with different types of cancer cells, i.e., SCC090 HNSCC cells, HCC78, and HCC827 lung cancer cells, SKBR3 breast cancer cells, and LNCaP prostate cancer cells (n = 3).

Subsequently, we optimized the HNSCC CTC recovery yield following enzyme-triggered release by *Sma*I. As shown in the workflow in Fig. 5A, CTCs were captured on the surface of the TDN-NanoGold Click Chip with the optimized conditions determined in the experiments in Fig. 4, then released after enzyme-triggered specific cleavage of TDN by *Sma*I. As described in the previous experiment for the optimization of CTC capture yield, an RT-ddPCR assay was utilized to quantify the copies of E6 transcripts in the artificial samples after the enzyme-triggered release of CTCs. The recovery yields for SCC090 HNSCC cells were calculated by Eq. (2) throughout the optimization process:

(2) $$\mathrm{Recovery yield}=E6\;{\mathrm{transcripts}}_{\mathrm{recovered}}/E6\;{\mathrm{transcripts}}_{\mathrm{spiked}}$$

First, we confirmed that the use of restriction endonuclease, *Sma*I (80 U/chip), was preferable for enzyme-triggered CTC release compared to the use of DNA endonuclease, e.g., DNase I (60 U/chip), as shown in Fig. S6. We calculated the recovery yield and viability of CTCs recovered by *Sma*I-triggered and DNase I-triggered releases. The results showed that the sequence-specific *Sma*I-triggered cleavage of TDN retained significantly higher viability of the recovered CTCs (viability: 76.0 ± 6.5%) in contrast to that (viability: 54.0 ± 8.1%) observed for the non-specific DNase I-triggered cleavage of TDN (Fig. 5B). Previous studies have shown that CuSO_4_/Cu^2+^ leads to minimal loss of viability even under co-culture over several days at the concentration of ~100 mM. [41,42] In this study, we verified the high cell viability of recovered CTCs from TDN-NanoGold Click Chips by successfully culturing them for over four days (Fig. 5C). The results demonstrated that the short exposure time (30 min) and low dose of CuSO_4_ (0.1 mM) would minimally impact CTCs’ viability. We then examined the recovery yield according to the varying amounts of *Sma*I (0, 20, 40, 60, 80, and 100 U/chip) and found that 80 U/chip resulted in the most robust enzyme-triggered release of CTCs with a recovery yield of 83.3 ± 6.1% (Fig. 5D).

After the optimization of CTC capture and recovery yields, we tested the general applicability of the TDN-NanoGold Click Chips for the detection of CTCs included in various artificial samples prepared by spiking other types of cancer cells, including HCC78 and HCC827 lung cancer cells, SKBR3 breast cancer cells, and LNCaP prostate cancer cells, into PBMCs isolated from whole blood of a healthy donor (2 mL). The capture/recovery yields were then calculated by CTC enumeration. The results demonstrated that TDN-NanoGold Click Chips can achieve capture and recovery yields ranging from 79.1% to 91.3% and from 73.2% to 83.3%, respectively, for all cancer cell types studied in this experiment (Fig. 5E).

### CTC Enumeration and quantification of CTC-derived E6/E7 transcripts in HNSCC patients

Under the optimal conditions identified above, we explored the clinical utility of TDN-NanoGold Click Chips in HNSCC for CTC-derived E6/E7 transcript quantification and CTC enumeration, including total HNSCC CTCs and subpopulation of P16(+) HNSCC CTCs, in 14 peripheral blood samples that were collected from 11 HPV(+) HNSCC patients over the course of clinical standard treatments. The HPV status of these 11 HNSCC patients was confirmed by P16 protein expression using IHC staining in formalin-fixed paraffin-embedded (FFPE) tissues.

**Fig. 6.**
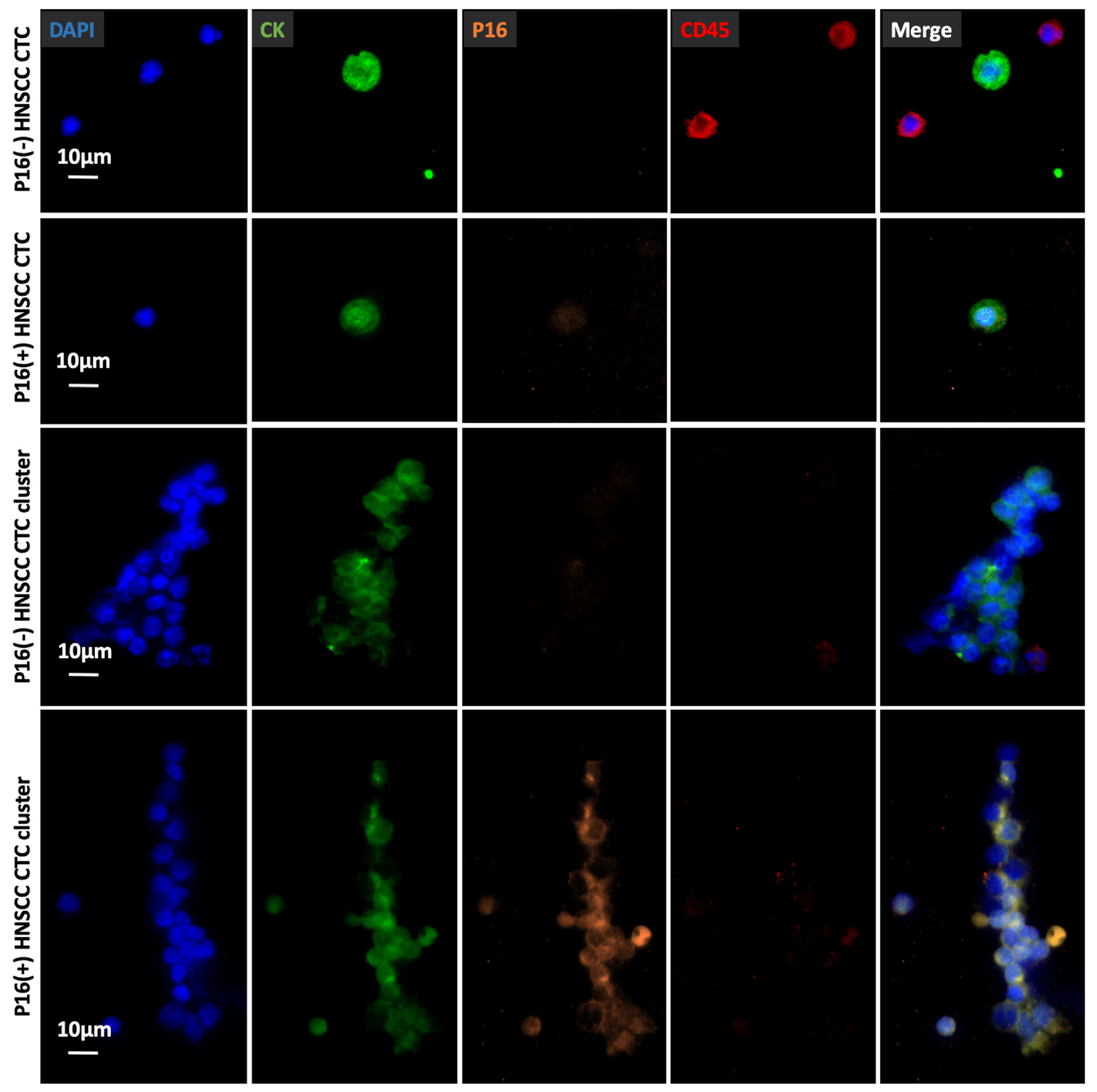
Representative micrographs of an immunocytochemistry (ICC)-stained P16(−) HNSCC CTC, P16(+) HNSCC CTC, 16(−) HNSCC CTC cluster, and a P16(+) HNSCC CTC cluster on TDN-NanoGold Click Chips from different HNSCC patients. Blue: DAPI stained nuclei; green: FITC stained CK; orange: TRITC stained P16; red: CY5 stained CD45. Scale bar, 10 μm. Data are representatives of five independent assays.

We performed immunofluorescent staining of P16 protein and cytokeratin (CK) for CTC identification. The immunofluorescent staining including CK, P16, CD45, and DAPI for P16(+) HNSCC CTC identification was performed using HPV16(+) SCC090 cells and HPV(−) HD PBMCs. SCC090 cells displayed a CK+ /P16 + /CD45-staining pattern, while the control PBMCs only have CD45 positivity, as shown in Fig. S7. Representative fluorescent microscopy images of a P16(−) HNSCC CTC, a P16(+) HNSCC CTC, a P16(−) HNSCC CTC cluster and a P16(+) HNSCC CTC cluster captured on TDN-NanoGold Click Chips from different HNSCC patients are shown in Fig. 6. The general applicability of TDN-NanoGold Click Chips in other cancers was also tested. Representative fluorescent microscopy images of CTCs captured from lung cancer, breast cancer, and prostate cancer are presented in Fig. S8, and CTC enumeration is summarized in Table S2.

**Fig. 7.**
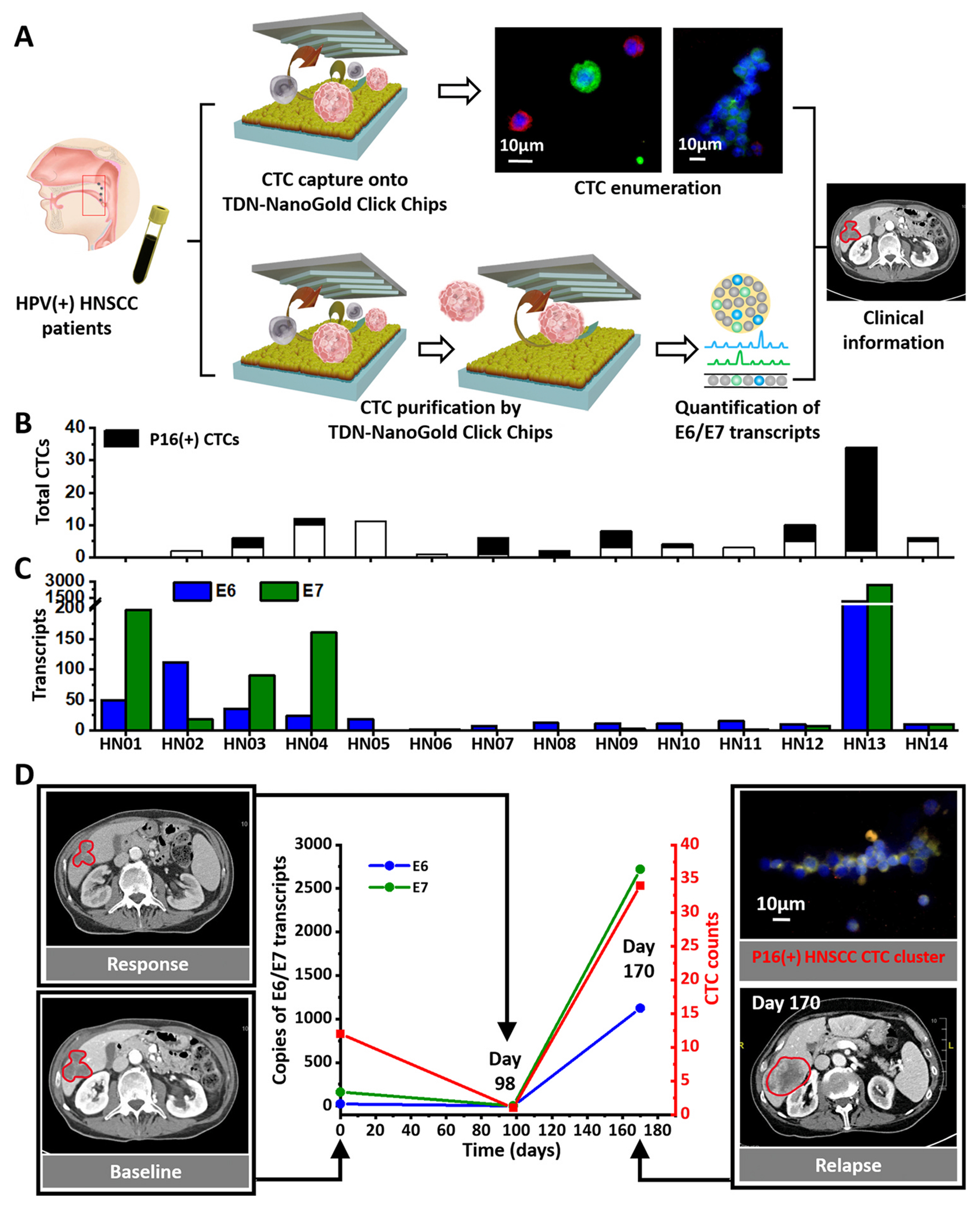
Clinical application of TDN-NanoGold Click Chips in HPV (+) HNSCC patients. TDN-NanoGold Click Chips were used for both CTC enumeration and quantification of HNSCC CTC-derived E6/E7 transcripts in HPV(+) HNSCC patients over the course of clinical standard treatments. A) Schematic illustration of the general workflow developed for conducting CTC enumeration and HNSCC CTC-derived E6/E7 transcript quantification. B-C) The HNSCC CTC counts, P16(+) HNSCC CTC counts, and HNSCC CTC-derived E6/E7 transcripts were detected in 14 blood samples from HNSCC patients. D) The dynamic changes (0–170 days) of P16(+) HNSCC CTC counts and HNSCC CTC-derived E6/E7 transcripts (per 2 mL blood) were observed for an HPV (+) HNSCC patient over the course of clinical standard treatments. Computed tomography (CT) images of this patient’s liver metastasis taken at baseline (Day 0), treatment response (Day 98), and relapse (Day 170). A P16(+) HNSCC CTC cluster was observed in the blood sample at Day 170.

The P16 protein status in CTCs as well as CTC-derived E6/E7 transcripts were analyzed among the 14 blood samples collected from 11 HPV(+) HNSCC patients (Fig. 7A). P16(+) HNSCC CTCs were detected in 9 out of the 14 samples (Fig. 7B). The reason for the remaining 5 samples with discordant P16 protein status may be attributed to the change in CTC count over the period between tissue biopsy and blood draw or caused by the underlying tumor heterogeneity. Meanwhile, HNSCC CTC-derived E6/E7 transcripts were detected from all 14 blood samples. Fig. 7C and Table S3 summarize the quantification of HNSCC CTC-derived E6/E7 transcripts in 14 blood samples from the 11 HPV(+) HNSCC patients. Overall, the detection rate for CTC-derived E6/E7 transcripts (14 out of 14) is higher than that for the P16(+) sub-population of CTCs (9 out 14) from HPV (+) HNSCC patients.

We monitored the dynamic changes in one HNSCC patient during treatment by aligning the P16(+) HNSCC CTC count and copies of HNSCC CTC-derived E6/E7 transcripts with CT images taken throughout clinical standard treatments. Serial blood samples were collected at Day 0 (baseline), and 98 and 170 days post-treatment. The corresponding P16(+) HNSCC CTC count and copies of HNSCC CTC-derived E6/E7 transcripts at each time point are plotted in Fig. 7D. At the last blood draw (170 days post-treatment), liver metastasis was detected by the CT, a clinical finding consistent with the dynamic changes in increased P16(+) HNSCC CTC count and copy numbers of HNSCC CTC-derived E6/E7 transcripts. It is noteworthy that a P16(+) HNSCC CTC cluster was observed in the last blood draw. The presence of CTC clusters often correlates with a more aggressive disease progression [43].

## Conclusion

In this study, we developed a top-down fabrication method for the preparation of TDN-NanoGold substrates via the hierarchical integration of three functional constituents, i.e., macroscale glass slides, sub-microscale self-organized NanoGold, and nanoscale self-assembled TDN. Our results revealed that all three functional constituents in the TDN-NanoGold substrates are essential to achieving enhanced cell-capture performance. The top-down fabrication method presented in this study introduces a simple and effective alternative to the fabrication methods previously developed for existing nanostructure-embedded cell-capture substrates [9], which primarily rely on direct deposition of different nanomaterials, e.g., nanowires, nanofibers, nanoparticles, and nanosheets, onto flat substrates. The TDN-NanoGold substrates were then assembled with microfluidic chaotic mixers. In conjunction with the use of CuAAC-mediated CTC capture and restriction enzyme-triggered CTC release, the resultant TDN-NanoGold Click Chips enable enumeration, purification, and molecular characterization of HNSCC CTCs. In contrast to the Tz-TCO Click Chemistry-mediated cell capture, the azide/alkyne motifs used in CuAAC-mediated CTC capture are relatively stable [44] yet sufficiently reactive in the presence of Cu (I) generated in situ, offering the desired shelf life of TDN-NanoGold Click Chips and alkyne-labeled capture antibody. We demonstrated the feasibility of applying TDN-NanoGold Click Chips in HPV(+) HNSCC for (i) counting HNSCC CTCs along with their sub-population with P16 protein expression, and (ii) quantifying CTC-derived E6 and E7 oncogenes, characteristic mRNA markers of the purified HNSCC CTCs, by RT-ddPCR.

This specific quantification capability of combining both conventional CTC enumeration and digital counting of CTC-derived E6 and E7 oncogenes was exploited to monitor the disease progression and treatment response. The quantification results of CTC enumeration and CTC-derived E6 and E7 oncogenes correlate well with the radiographic imaging and clinical outcomes. Compared to HNSCC CTC counts, the copy numbers of CTC-derived E6/E7 transcripts showed higher sensitivity and a broader dynamic range. On top of the CTCs’ unique specificity in circulation (only present in epithelial cells), CTC-derived E6/E7 transcripts enable more sensitive and specific detection of systemic tumor burden in patients with HPV(+) HNSCC. These results may enforce more informed and precise therapeutic decisions for further stratifying HPV(+) HNSCC patients in both clinical trials and clinical practice. Notably, HPV-driven oropharyngeal HNSCC has a favorable outcome after multi-modality treatment. However, long-term treatment side effects, including dry mouth and trouble swallowing, pose as treatment challenges. Therefore, there has been increasing interest in less intense treatment to preserve patients’ quality of life. [45,46] Non-invasive and accurate detection of HPV(+) HNSCC CTCs and CTC-derived E6/E7 oncogenes in peripheral blood is a potentially useful way to offer enhanced prognostication and response assessment. Due to the relatively low incidence of HPV (+) HNSCC, our clinical validation study is conducted in a patient cohort with small sample size in this proof-of-concept study. Further validation of TDN-NanoGold Click Chips in a larger group of HPV(+) HNSCC patients will be required in the future.

## Experimental section

### Preparation of tetrahedral DNA nanostructure (TDN)

The single-strand DNA (ssDNA) was from Shanghai Sangon Biological Engineering Technology and Services Co. Ltd. (Shanghai, China).

NH_2_-labeled TDN was prepared by mixing 1 μL of 100 μM strands 1–5 with 86 μL of TM buffer and 10 μL of TCEP solution (30 mM) (Table S1), and biotinylated TDN was prepared using strand 1, strand 2, strand 3, strand 4 and strand 6. The mixture was heated to 95 °C for 10 min, and then cooled to 4 °C within 30 s using BioRad S1000 Thermal Cycler (Hercules, CA). To serve as a control group, a double-strand DNA (dsDNA) was prepared by the same procedure.

### Preparation of NanoGold substrates

Frosted glass slides were immersed in 3 mM HAuCl_4_, followed by adding ammonium hydroxide (20 μL per 1 mL of HAuCl_4_ solution). Reaction solution was immediately mixed for 1 min. Then, the slides were washed with deionized water (ddH_2_O) and incubated with 1 mM NaBH_4_ solution for 1 min. After the washing steps, the slides were immersed in a mixed solution of HAuCl_4_ and NH_2_OH·HCl (1:1, molar ratio), shaken for 2 min and reacted for 13 min at room temperature to accomplish the growth step. After being washed with ddH_2_O for three times, the slides were dried under nitrogen for future use.

### Fabrication of azide-grafted TDN-NanoGold substrates

The prepared NanoGold substrates were immersed in a self-assembled NH_2_-labeled TDN solution of variable concentrations and incubated at room temperature for 2 h, resulting in TDN-NanoGold substrates. The TDN-NanoGold substrates with terminal amine groups were incubated in NHS-PEG-N_3_ PBS solution (2 mM, pH=8.5) for 1 h at room temperature. After being washed 3 times by PBS and ddH_2_O, azide-grafted TDN-NanoGold substrates (TDN-NanoGold Click Chips) were dried by nitrogen blow and then stored at − 20 °C before use.

In addition, for TDN-NanoGold substrates characterization, a GNPs-grafted TDN-NanoGold substrate was prepared to visualize the TDN on the NanoGold substrate. A biotinylated TDN-NanoGold substrate was prepared by immersing a NanoGold substrate in a self-assembled biotinylated TDN solution and incubated at room temperature for 2 h, followed by another incubation with streptavidin-labeled gold nanoparticles (GNPs, 10 nm, 0.2 mg mL^−1^) for 30 min at room temperature. A Bruker atomic force microscope (AFM) was employed to measure the roughness of the GNPs-grafted TDN-NanoGold substrate.

### Fluorescence characterization of azide-grafted TDN-NanoGold substrates

To demonstrate the feasibility and specificity of bioorthogonal ligation between azide and alkyne motifs, Cy5-labeled alkyne reagent (2.0 mM, Click Chemistry Tools) in PBS was used to treat azide-grafted TDN-NanoGold substrates as well as an H_2_N-labeled TDN-NanoGold substrate (as a control) for 30 min. Unbound Cy5-labeled alkyne was washed off with deionized water five times prior to characterization. To investigate the restriction enzyme cleavage, the Cy5-grafted TDN-NanoGold substrates were treated with *Sma*I (80 U) in PBS (200 μL) for 30 min, followed by being washed with water five times. Fluorescence images were captured with a fluorescence microscope (Nikon 90i).

### Preparation of alkyne-anti-EpCAM conjugate

Alkyne-anti-EpCAM conjugate was prepared by incubating Alkyne-PEG4-NHS ester (0.5 mM, Click Chemistry Tools) with human EpCAM/TROP-1 antibody (Goat IgG, 0.5 μg μL^−1^, R&D Systems, Inc.) in PBS at room temperature for 30 min. The alkyne-anti-EpCAM conjugate was diluted to 50 ng/μL and stored at − 20 °C.

### CTC capture and release in TDN-NanoGold Click Chips

The artificial CTC samples were prepared by spiking 50 SCC090 cells into PBMCs isolated from 2 mL HD whole blood. Either the artificial CTC samples or clinical PBMC samples (see section below) were incubated with alkyne-anti-EpCAM (50 ng) in 200 μL PBS at room temperature for 30 min and then centrifuged at 300 g for 3–10 min to remove the excess alkyne-anti-EpCAM. The resulting alkyne-grafted CTC samples were then purified using TDN-NanoGold Click Chips.

For CTC capture, the alkyne-grafted artificial CTC samples and HNSCC patient PBMC samples (200 μL) were injected into TDN-NanoGold Click Chips at the designed conditions. For CTC enumeration, the captured HNSCC CTCs were fixed with 2.0% formaldehyde and stained for fluorescence imaging. To release CTCs, 200 μL of *Sma*I with different amounts was injected into the Click Chip system at a flow rate of 0.5 mL/h, then 100 μL of PBS was injected to recover the released CTCs, followed by subsequent CTC enumeration or molecular analysis using RT-ddPCR.

### Collection and treatment of HNSCC patients’ blood samples

We enrolled 14 whole blood samples from 11 HNSCC patients from October 2019 to June 2021. Patients who had other uncontrolled malignant tumors, uncontrolled infection, Mycobacterium tuberculosis, or severe mental disease were excluded. Peripheral venous blood samples were collected at diagnosis or serially over the course of treatment with the written informed consent of these patients for this study according to the IRB protocol (IRB #10–001785) at UCLA. Each 8.0 mL blood sample was collected in a BD Vacutainer glass tube (BD Medical). For each HNSCC patient sample, three 2.0 mL vials of blood were separately centrifuged to collect peripheral blood mononuclear cells (PBMCs). Two of the PBMC samples were used for CTC capture for total HNSCC CTC enumeration and P16(+) HNSCC CTC enumeration, respectively. In parallel, another 2.0-mL blood sample was used for CTC release and HNSCC CTC E6/E7 transcript quantification by RT-ddPCR.

### Immunostaining and identification of HNSCC CTCs from HNSCC patients’ blood samples

After CTC capture using TDN-NanoGold Click Chips in patients’ blood samples, the CTC samples were fixed by 4% PFA and then subjected to a four-color ICC staining [47] with DAPI, anti-CK, anti-P16 [48,49], and anti-CD45 for identification of total HNSCC CTCs and P16(+) HNSCC CTCs. The substrates were then scanned with a fluorescence microscope (Nikon 90i). An automatic scan was carried out under 20 × magnification with DAPI, FITC, TRITC, and Cy5 channels corresponding to nuclear, CK, P16, and CD45 staining, respectively. When analyzing the multi-channel ICC micrographs, HNSCC CTCs (DAPI+ /CK+ /P16− /CD45−), P16(+) HNSCC CTCs (DAPI+ /CK + /P16+ /CD45−) and WBCs (DAPI+ /CK− /P16− /CD45+) were identified with intact nuclear morphology. HNSCC CTC clusters were defined as aggregated HNSCC CTCs with at least two HNSCC CTCs attached to each other.

For CTC samples from other cancer patients, a three-color ICC staining was employed and the captured cells of DAPI+ /CK+ /CD45-with intact nuclear morphology were identified as the CTCs.

### RNA extraction and RT-ddPCR detection of E6/E7 transcripts in HNSCC CTCs

The CTCs released from TDN-NanoGold Click Chips were lysed by TRI Reagent for RNA extraction using a Direct-zol^™^ RNA MicroPrep Kit (ZYMO Research Corp.) according to the manufacturer’s protocol. The purified RNA was reverse-transcribed to complementary DNA (cDNA) using a Thermo Scientific Maxima H Minus Reverse Transcriptase Kit according to the manufacturer’s instructions. Four microliter of cDNA products were then pre-amplified using a Prelude PreAmp Master Mix Kit to obtain 10 μL pre-amplified product according to the manufacturer’s instructions of (Takara Bio). For ddPCR, the reaction mixture (20 μL) including 2 μL of the pre-amplified product was loaded into each well. Data were analyzed using the QuantaSoft^™^ software package to calculate the copy numbers of E6/E7 transcripts detected from individual samples.

## Supplementary Material

Hierarchical integration of DNA nanostructures and NanoGold onto a microchip facilitates covalent chemistry-mediated purification of circulating tumor cells in head and neck squamous cell carcinoma-SM

## Data Availability

Data will be made available on request.
