# Supplementary material for: Hierarchical integration of DNA nanostructures and NanoGold onto a microchip facilitates covalent chemistry-mediated purification of circulating tumor cells in head and neck squamous cell carcinoma
Source: Nano Today. Author manuscript; Available in PMC 2023 Nov 30. (PMC10688595; doi:10.1016/j.nantod.2023.101786)
Supplement: Hierarchical integration of DNA nanostructures and NanoGold onto a microchip facilitates covalent chemistry-mediated purification of circulating tumor cells in head and neck squamous cell carcinoma-SM [file NIHMS1945169-supplement-Hierarchical_integration_of_DNA_nanostructures_and_NanoGold_onto_a_microchip_facilitates_covalent_chemistry-mediated_purification_of_circulating_tumor_cells_in_head_and_neck_squamous_cell_carcinoma-SM.docx]

**Supplementary information**

Hierarchical integration of DNA nanostructures and NanoGold onto a microchip facilitates covalent chemistry-mediated purification of circulating tumor cells in head and neck squamous cell carcinoma

Na Sun,^1,2,†^ Ceng Zhang,^1,3,†^ Jing Wang,^1,4,†^ Xinmin Yue,^5^ Hyo Yong Kim,^1^ Ryan Y. Zhang,^1^ Hongtao Liu,^1,6^ Josephine Widjaja,^1^ Hubert Tang,^1^ Tiffany X. Zhang,^1^ Jinglei Ye,^1^ Audrey Qian,^1^ Chensong Liu,^1^ Alex Wu,^1^ Katharina Wang,^1^ Michael Johanis,^1^ Peng Yang,^1^ Honggang Liu,^4^ Meng Meng^1,5,^* Li Liang,^7,8,^* Renjun Pei,^2,^* Wanxing Chai-Ho,^9,^* Yazhen Zhu,^1,10,11,^* Hsian-Rong Tseng^1,10,^*

^1^California NanoSystems Institute, Crump Institute for Molecular Imaging, Department of Molecular and Medical Pharmacology, University of California, Los Angeles, Los Angeles, CA 90095, USA

^2^Key Laboratory for Nano-Bio Interface, Suzhou Institute of Nano-Tech and Nano-Bionics, University of Chinese Academy of Sciences, Chinese Academy of Sciences, Suzhou 215123, P.R. China

^3^Department of Pathology, School of Basic Medical Sciences, Southern Medical University, Guangzhou, 510515, P.R. China

^4^Department of Pathology, Beijing Tongren Hospital, Capital Medical University, Beijing, 100730, China

^5^College of Pharmacy, State Key Laboratory of Medicinal Chemical Biology and Tianjin Key Laboratory of Molecular Drug Research, Nankai University, Tianjin 300353, P.R. China

^6^Department of Pathology, The First Affiliated Hospital of Shandong First Medical University & Shandong Provincial Qianfoshan Hospital, Shandong, 250014, P.R. China

^7^Department of Pathology, Nanfang Hospital and School of Basic Medical Sciences, Southern Medical University, Guangzhou 510515, P.R. China

^8^Guangdong Province Key Laboratory of Molecular Tumor Pathology, Guangzhou 510515, Guangdong Province, P.R. China

^9^Department of Medicine, Division of Hematology/Oncology, David Geffen School of Medicine, University of California, Los Angeles, Los Angeles, CA 90095, USA.

^10^Jonsson Comprehensive Cancer Center, University of California, Los Angeles, Los Angeles, CA 90095, USA

^11^Department of Pathology and Laboratory Medicine, David Geffen School of Medicine, University of California, Los Angeles, Los Angeles, CA 90095, USA.

†N. S., C. Z. and J.W. contributed equally.

*To whom correspondence should be addressed. e-mail: [hrtseng@mednet.ucla.edu](mailto:hrtseng@mednet.ucla.edu) (H.-R.T.); [lli@smu.edu.cn](mailto:lli@smu.edu.cn) (L.L.); [rjpei2011@sinano.ac.cn](mailto:rjpei2011@sinano.ac.cn) (R.P.); [wchaiho@mednet.ucla.edu](mailto:wchaiho@mednet.ucla.edu) (W.C.); [mengmeng@nankai.edu.cn](mailto:mengmeng@nankai.edu.cn) (M.M.); [yazhenzhu@mednet.ucla.edu](mailto:yazhenzhu@mednet.ucla.edu) (Y.Z.)

**Fig. S1.** Fabrication of NanoGold substrate.

**Fig. S2.** Preparation and characterization of teterhedral DNA nanostructure (TDN).

**Fig. S3.** Specific cleavage of target TDN sequence by SmaI.

**Fig. S4**. Preparation of Alkyne-conjugated anti-EpCAM antibodies.

**Fig. S5.** SEM images of captured cells on the TDN-NanoGold substrate (A, B) and the flat substrate (C), respectively.

**Fig. S6.** Optimization of the concentrations for SmaI (A) and DNase (B) on Cy5-labeled TDN-NanoGold substrate according to the fluorescence decrease ratio (F/F_0_).

**Fig. S7**. Immunofluorescent staining of CK, P16, CD45 and DAPI for HPV16(+) SCC090 cells and HPV(-) HD PBMCs.

**Fig. S8**. Representative fluorescent microscopy images of CTCs captured from blood samples of patients with lung cancer, breast cancer and prostate cancer.

**Table S1.** DNA sequences used in this study.

**Table S2.** CTC enumeration of blood samples from patients having lung cancer, breast cancer and prostate cancer.

**Table S3.** Clinical information of head and neck cancer patients.


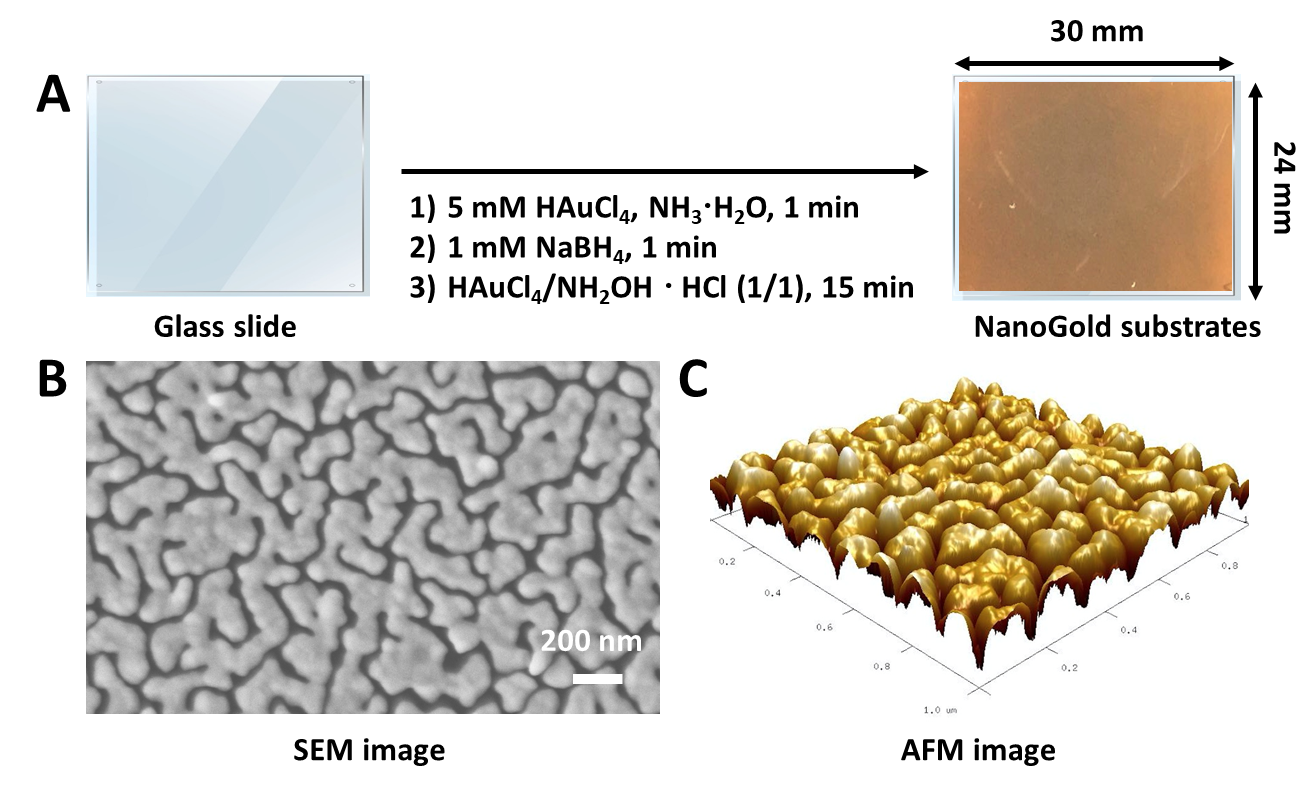


**Fig. S1. Fabrication of NanoGold substrate. A)** NanoGold substrate was formed by introducing self-organized NanoGold structures onto a glass substrate (30 × 24 mm^2^) via a gold seeding approach. **B)** scanning electron microscope (SEM) and **C)** atomic force microscope (AFM) images for the characterization of NanoGold substrate.


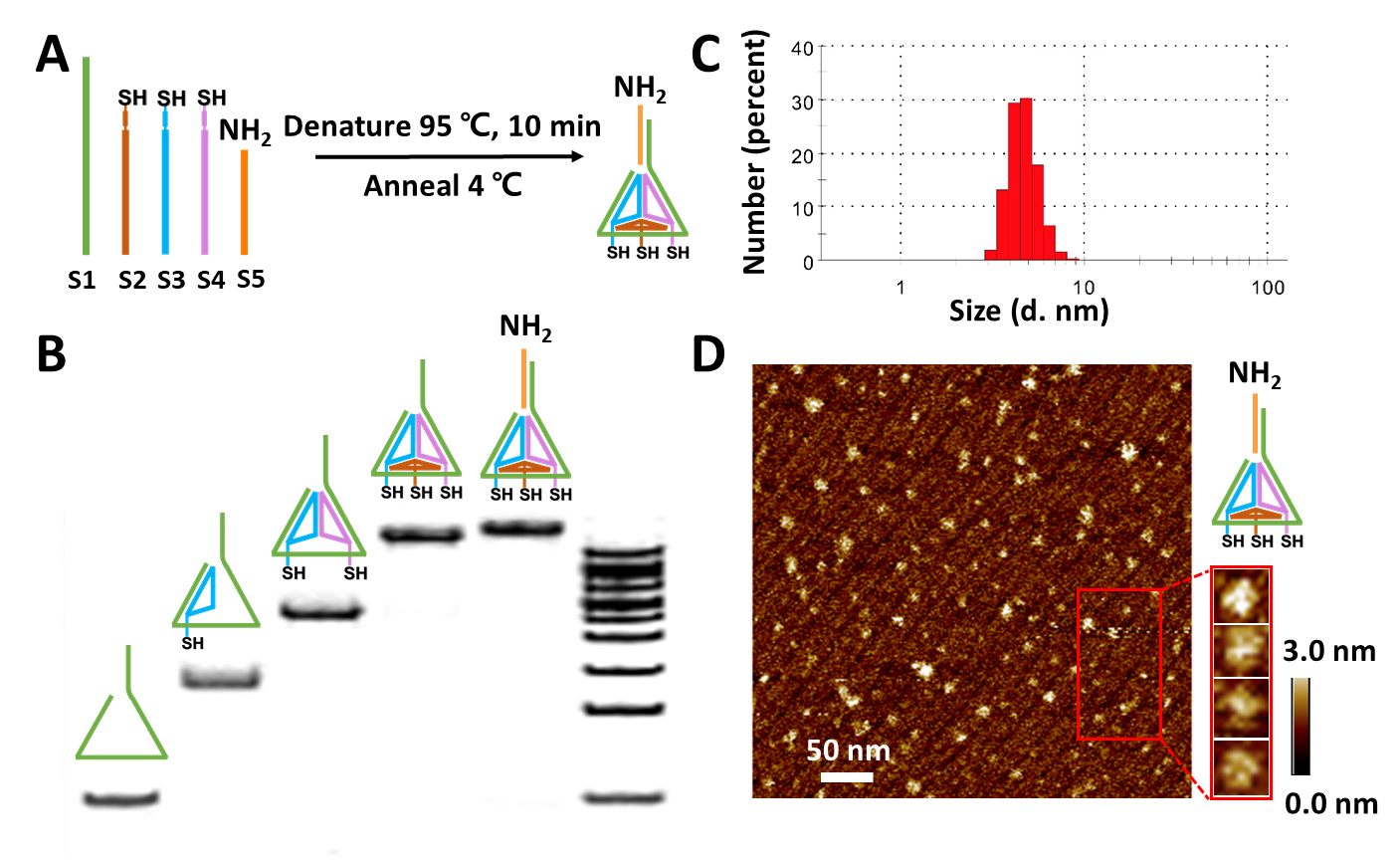


**Fig. S2. Preparation and characterization of teterhedral DNA nanostructure (TDN). A)** Conjugation of TDN was conducted by simply mixing all DNA strands, followed by denaturation at 95 ℃ for 10 min and annealing at 4 ℃. **B)** Step-by-step TDN assembly was demonstrated by polyacrylamide gel electrophoresis (PAGE). **C)** Dynamic light scattering (DLS) and **D)** AFM analyses confirmed that the conjugated TDN has uniform size (5.8 nm).


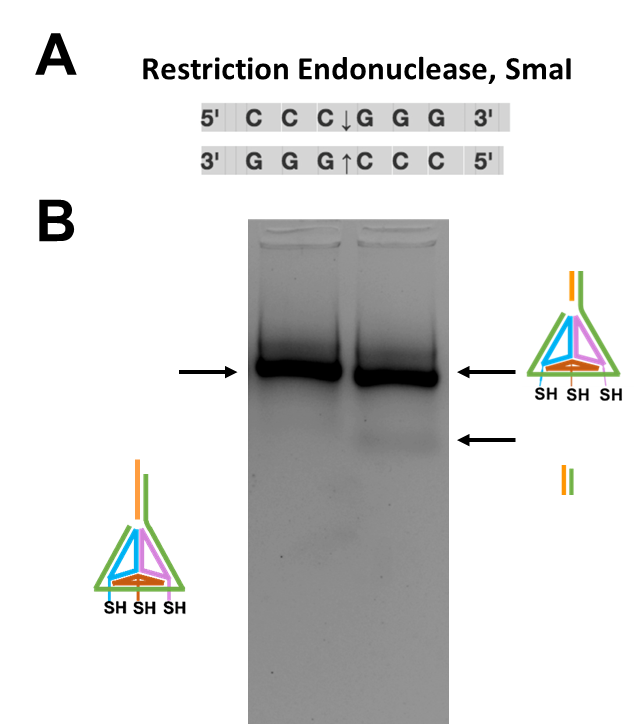


**Fig. S3. Specific cleavage of target TDN sequence by SmaI. A)** Sequence information and cleavage site for SmaI. **B)** Enzyme-triggered cleavage of target TDN sequence by SmaI was confirmed by PAGE.


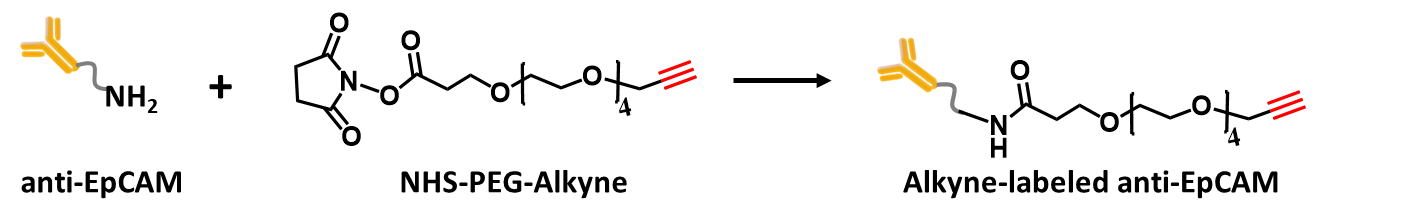


**Fig. S4. Preparation of Alkyne-conjugated anti-EpCAM antibodies.**


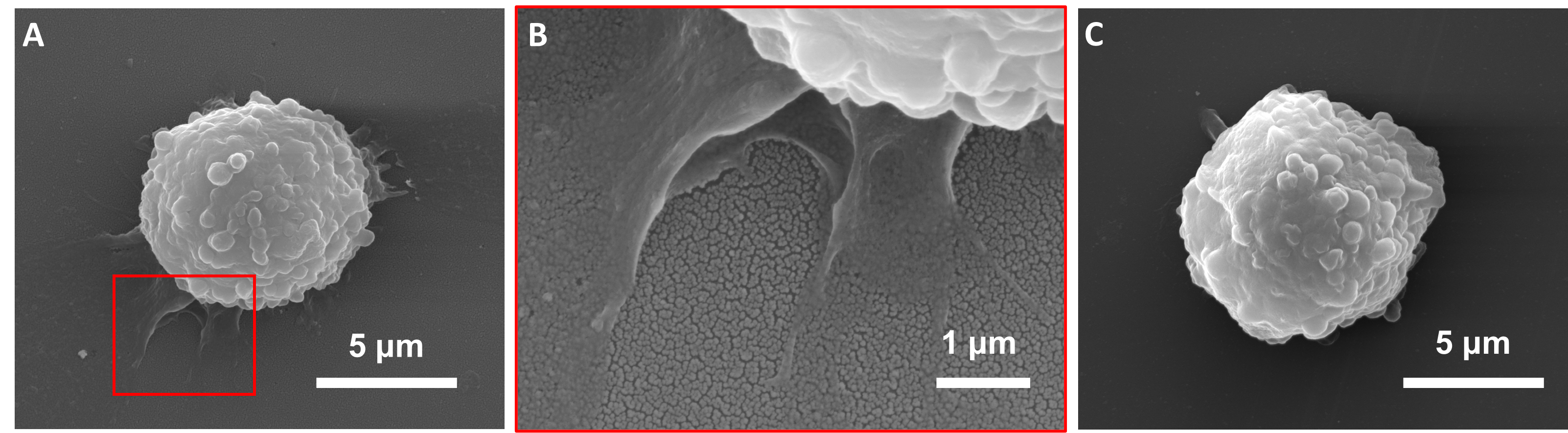


**Fig. S5. SEM images of captured cells on the TDN-NanoGold substrate (A, B) and the flat substrate (C), respectively.**


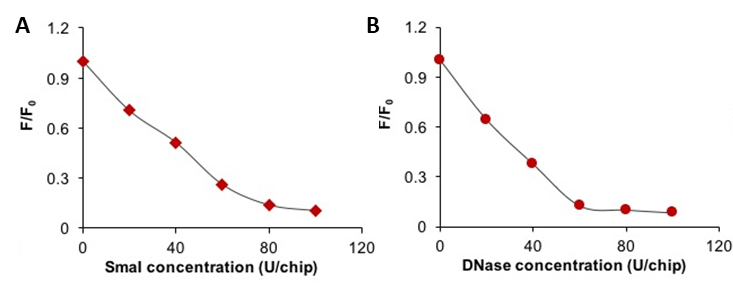


**Fig. S6.** **Optimization of the concentrations for SmaI (A) and DNase (B) on Cy5-labeled TDN-NanoGold substrate according to the fluorescence decrease ratio (F/F_0_).**


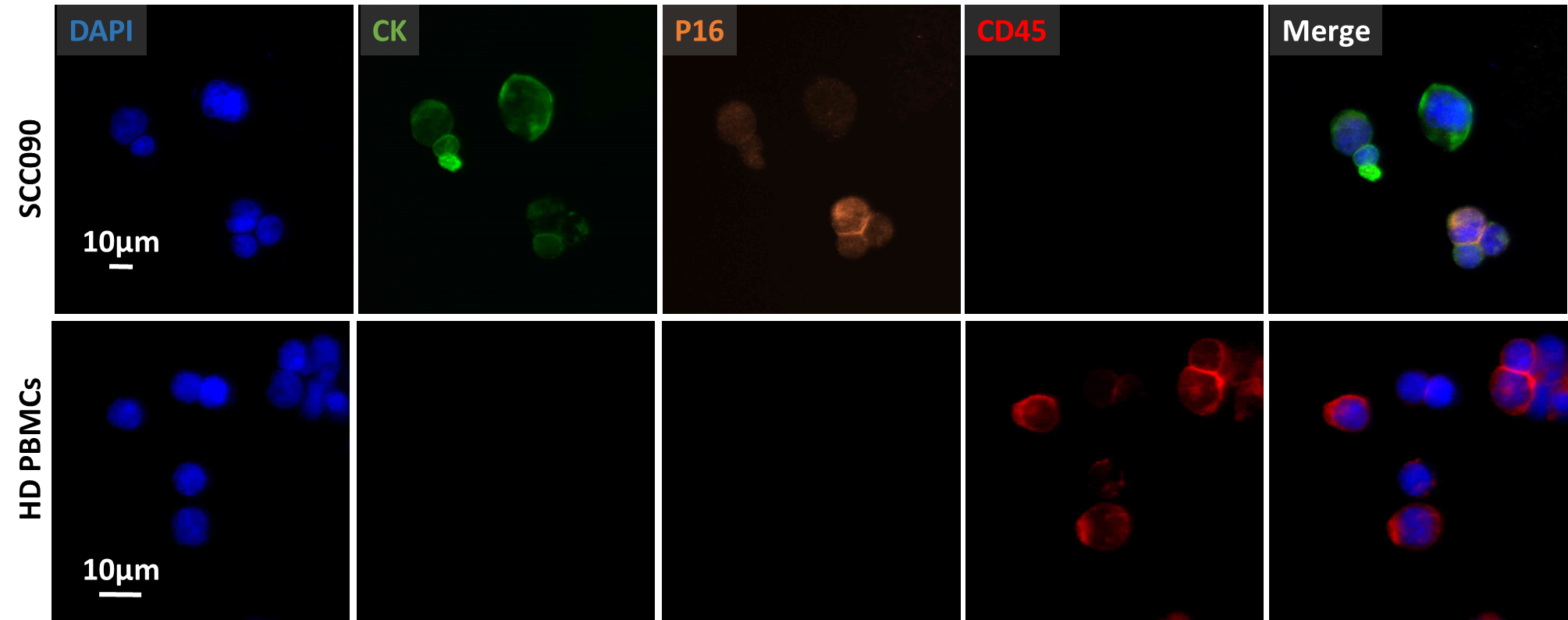


**Fig. S7**. **Immunofluorescent staining of CK, P16, CD45 and DAPI for HPV16(+) SCC090 cells and HPV(-) HD PBMCs.**


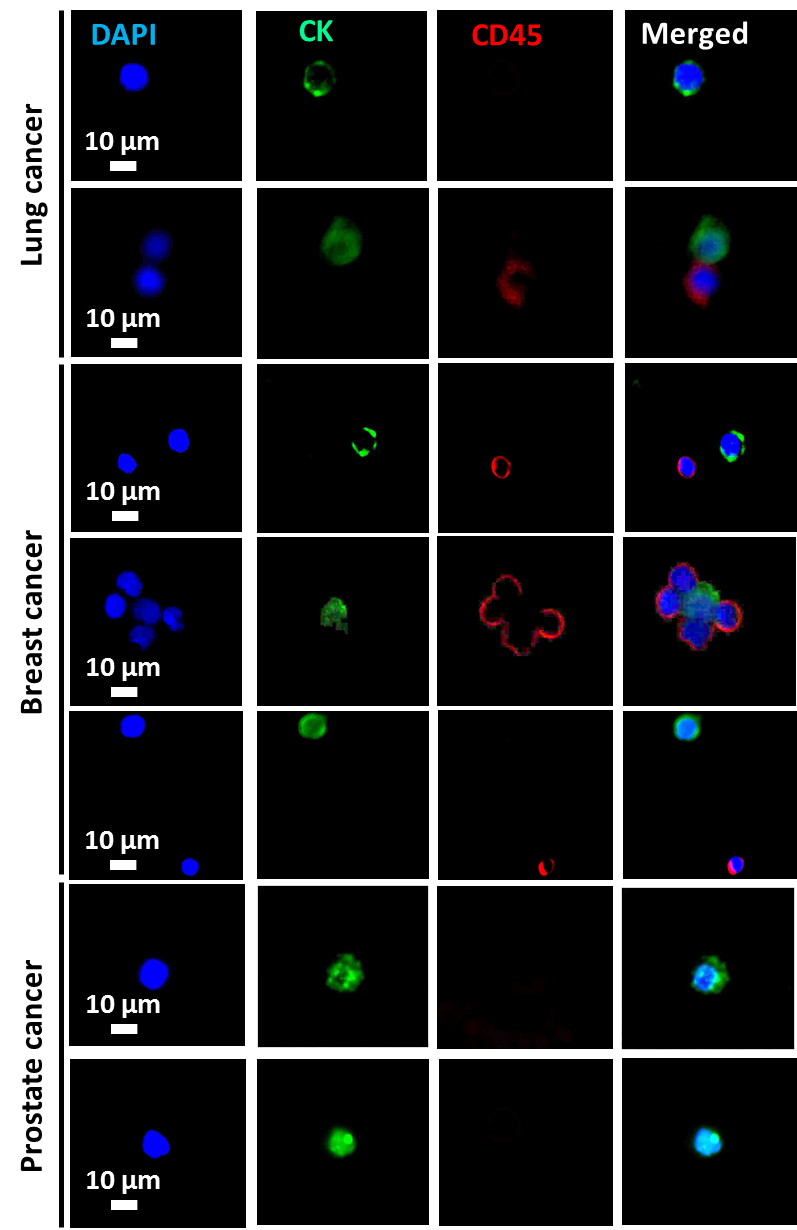


**Fig. S8**. Representative fluorescent microscopy images of CTCs captured from blood samples of patients with lung cancer, breast cancer and prostate cancer.

**Table S1. DNA sequences used in this study.**

| **Group** | **Strand** | **Sequence (5’ - 3’)** |
| --- | --- | --- |
| TDN | Strand 1 | ACATTCCTAAGTCTGAAACATTACAGCTTGCTACACGAGAAGAGCCGCCATAGTATTTTTTTTTT**GTATCCCC^GGGCTCA** |
|  | Strand 2 | HS-TATCACCAGGCAGTTGACAGTGTAGCAAGCTGTAATAGATGCGAGGGTCCAATAC |
|  | Strand 3 | HS-TCAACTGCCTGGTGATAAAACGACACTACGTGGGAATCTACTATGGCGGCTCTTC |
|  | Strand 4 | HS-TTCAGACTTAGGAATGTGCTTCCCACGTAGTGTCGTTTGTATTGGACCCTCGCAT |
|  | Strand 5 | NH_2_-TTTTT**TGAGCCC^GGGGATAC** |
| dsDNA | Strand 5 | NH_2_-TTTTT**TGAGCCC^GGGGATAC** |
|  | Strand 6 | HS-TTTTTTTTTT**GTATCCCC^GGGCTCA** |
| - The complementary sequences containing enzyme-triggered cleavage site by SmaI are bolded. - The enzyme-triggered cleavage site by SmaI is underlined. | | |

**Table S2. CTC enumeration of blood samples from patients having lung cancer, breast cancer and prostate cancer.**

| **Patients** | **Gender** | **Age (Y)** | **Clinical stage** | **Histology subtype** | **CTC counts**  **(per 1 mL blood)** |
| --- | --- | --- | --- | --- | --- |
| Lung cancer  L-01  L-02 | F  M | 62  54 | III  IV | Non-small cell lung cancer  Small cell lung cancer | 6  8 |
| Breast cancer  B-01  B-02  B-03 | F  F  F | 51  48  46 | IV  IV  IV | Ductal carcinoma  Ductal carcinoma  Ductal carcinoma | 12  12  11 |
| Prostate cancer  P-01  P-02 | M  M | 50  55 | IV  IV | Adenocarcinoma  Adenocarcinoma | 8  7 |

**Table S3. Clinical information of head and neck cancer patients.**

| **Sample ID** | **Patient ID** | **Age** | **Sex** | **Primary tumor location** | **HPV/p16 status in tissue** | **Stage** | | | **Total CTCs** | **P16+ CTCs** | **E6** | **E7** |
| --- | --- | --- | --- | --- | --- | --- | --- | --- | --- | --- | --- | --- |
|  |  |  |  |  |  | **T** | **N** | **M** |  |  |  |  |
| HN01 | Pt01 | 59 | male | Oropharynx | HPV+/p16+ | T2 | N2 | M0 | 0 | 0 | 50 | 198 |
| HN02 | Pt02 | 67 | female | Oropharynx | HPV+/p16+ | T1 | N1 | M0 | 2 | 0 | 112 | 18 |
| HN03 | Pt03 | 61 | male | Oropharynx | HPV+/p16+ | T2 | N2 | M0 | 6 | 3 | 36 | 90 |
| HN04 | Pt04 | 70 | male | Oropharynx | HPV+/p16+ | T4 | N2 | M1 | 12 | 2 | 24 | 160 |
| HN05 | Pt05 | 60 | male | Oropharynx | HPV+/p16+ | T1 | N1 | M0 | 11 | 0 | 18.6 | 0 |
| HN06 | Pt04-2 | 70 | male | Oropharynx | HPV+/p16+ | T4 | N2 | M1 | 1 | 0 | 1.4 | 1.8 |
| HN07 | Pt01-2 | 59 | male | Oropharynx | HPV+/p16+ | T2 | N2 | M0 | 6 | 5 | 6.8 | 0 |
| HN08 | Pt06 | 69 | female | Tongue cancer | HPV+/p16+ | T3 | N0 | M0 | 2 | 2 | 12.4 | 0 |
| HN09 | Pt07 | 66 | female | Oral Cavity | HPV+/p16+ | T4a | N1 | M0 | 8 | 5 | 11.2 | 3 |
| HN10 | Pt08 | 65 | male | Tongue cancer | HPV+/p16+ | T2 | N0 | M0 | 4 | 1 | 11.2 | 0 |
| HN11 | Pt09 | 58 | male | Oropharynx | HPV+/p16+ | T2 | N0 | M0 | 3 | 0 | 15.6 | 1.4 |
| HN12 | Pt10 | 65 | male | Tongue cancer | HPV+/p16+ | T2 | N2c | M0 | 10 | 5 | 10.2 | 7.2 |
| HN13 | Pt04-3 | 70 | male | Oropharynx | HPV+/p16+ | T4 | N2 | M1 | 34 | 32 | 1124 | 2720 |
| HN14 | Pt11 | 76 | female | Oropharynx | HPV+/p16+ | T4a | N2b | M0 | 6 | 1 | 10 | 10 |
